# Unhealthy mind in a healthy body: A criticism to eliminativism in psychopathology

**DOI:** 10.3389/fpsyt.2022.889698

**Published:** 2022-09-30

**Authors:** Francesco Mancini, Alessandra Mancini, Cristiano Castelfranchi

**Affiliations:** ^1^Schools of Cognitive Psychotherapy (APC-SPC), Rome, Italy; ^2^Department of Psychology, Telematic University of Rome “Guglielmo Marconi”, Rome, Italy; ^3^Istituto di Scienze e Tecnologie della Cognizione - CNR, Rome, Italy

**Keywords:** reductionism, emergence, intentions, psychopathology, mind-body problem, mental processing

## Abstract

In this article we criticize the thesis “The diseases we treat are diseases of the brain”. A first criticism is against the eliminativist perspective and in favor of a perspective that is still reductionist but emergentist and functionalist. In a second part, we try to answer the question “under which conditions can we consider this statement legitimate?”. We argue that only those mental disorders whose neural substrate has clearly neuropathological characteristics, i.e., anomalies with respect to the laws of good neural functioning, can be considered “brain diseases.” We propose that it is not sufficient to observe a simple difference between the brains of people with psychopathology, that is, with anomalies with respect to the laws of good psychological functioning, and that of people without psychopathology. Indeed, we believe it is a categorical error to postulate a neuropathology starting from a psychopathology. Finally, we summarize some research that shows how purely psychological interventions can reduce or eliminate the differences between the brains of people with or psychopathology and those of people without.

## Introduction

In 2015, an editorial entitled *The Future of Psychiatry as Clinical Neuroscience*, was published on the important JAMA Psychiatry (1). The main argument concerned the increase in knowledge on the differences between the brains of people affected by a given psychopathology (e.g., depression) and those of people not affected or affected by a different psychopathology. “Technologic advances have enhanced our ability to study the brain, and new findings have reshaped the fundamental way in which we understand psychiatric illness. For example, although depression was once characterized as simply a monoaminergic deficit, new research is expanding our understanding of depression across multiple levels of analysis—from circuits, to neuro transmitters, to synaptic plasticity, to second messenger systems” (1). The conclusion was somewhat apodictic: “The diseases we treat are diseases of the brain” (1).

This sentence, beyond the real intentions of those who wrote it, lends itself well to discuss two theses, connected to each other, concerning psychopathology, and which, albeit with different nuances, can be glimpsed behind the so-called “biological psychiatry” (2). The first thesis is that psychopathology, like any other psychic event, is substantially reducible to neural events, *therefore* it should be described and explained *only* with neuroscientific concepts (i.e., eliminating the mental level of description). Following this approach, with neuroscientific advances, mentalistic concepts, still used today for describing, explaining and treating psychopathology, run the risk to be considered like phlogiston in chemistry (i.e., according to the “eliminative materialist”, the explanation in terms of *beliefs, desires*, and *fears*, will become obsolete, thanks to advancements in neuroscience) (3). In that, following this eliminativist thesis, psychotherapy and the psychological models of psychopathology on which it is based, would have no value.

The second thesis, connected with the previous one, argues that psychopathology, that is, mental illnesses, are actually “diseases of the brain.” These two theses deserve to be discussed in order to restore the right dignity to mentalist descriptions and explanations, and therefore also to strictly psychological interventions, without however falling back into the mind-brain dualism.

Therefore, in the first part of this article, we argue against eliminativism, and in favor of an emergentist approach which, while on the one hand considers the brain as the foundation of the mind and the mind as an expression of brain activity, on the other hand believes it necessary to adopt a multilayer, complex, self – emergent view of reality, that allows to give mental concepts the right role in the explanation of psychopathology.

Having ascertained the scientific legitimacy of using the mental level to explain psychopathology, in the second part of the article, we interrogate ourselves on what are the conditions for affirming or denying that “The diseases we treat are diseases of the brain”.

## Eliminativism and the emergentist criticism

Eliminativism is defined by the Dictionary of Philosophy of Mind: **“***The view that, because mental states and properties are items posited by a protoscientific theory (called folk psychology), the science of the future is likely to conclude that entities such as beliefs, desires, and sensations do not exist. The alternate most often offered is physicalist and the position is thus often called “eliminative materialism”* (4–7).

One of the most convincing criticisms to eliminativism comes from the so-called emergentism, which rejects the mind-brain dualism and accepts that the mind is a product of the brain, that any mental phenomenon corresponds to a neural phenomenon and that it cannot exist a mind without a brain or without its implementation in a material support. Emergentism, however, is different from eliminativism, because it assumes that the mind is an emergent phenomenon, or rather that mental phenomena are emergent features of complex brains (8) and thus they are not entirely reducible to it. They should be described and modeled at the macro-function layer implemented in- and emergent from the underlying micro-function of the material substrate. “A property of a system is said to be emergent if it is a new outcome of some other properties of the system and their interaction, while it is itself different from them” (9). As Chalmers put it “We can say that a high-level phenomenon is strongly emergent with respect to a low-level domain when the high-level phenomenon arises (in some sense) from the low-level domain, but truths concerning that phenomenon are not deducible even in principle from truths in the low-level domain (…). I think there is exactly one clear case of a strongly emergent phenomenon, and that is the phenomenon of consciousness” (10). However, strong epistemological emergence is different from ontological emergence, which rejects the layered model of reality as divided into a discrete hierarchy of levels (11).

At the basis of emergence there is the idea, ancient and shared by many, that the whole is greater than the sum of its parts. Nature (and, in nature, society) has different levels of structure, organization, dynamics, and “*functions*,” each macro-layer is grounded on the entities, properties and mechanisms of the lower layer (micro) but implies the emergence of macro-layer properties (12). For example, consider the concept of “Information” (that of Information Theory, Computer Science, etc.). It could be argued that “Information” is merely “energy.” Yes, but it is a specific level of energy dynamics and a *function* that energy assumes at a certain level of organization of the matter and of its processes. We could not eliminate the concept of “information,” because in nature there is only “energy.” However, is “information” nothing but energy? No, it is something more; it's energy with new characteristics, processes, laws; a new level of functions and effects, requiring their own “laws” and “concepts,” not meta-physical, but physical at a different level.

Nature organizes itself into emerging levels of complexity, with new structures, *which require their own scientific concepts and laws*, not existing at the micro level. Indeed, “Emergence occurs in *complex systems* in which novel properties emerge through the *aggregate functions* of the parts of that system” (8). As said, this holds even within the neural level: human experience and behavior are due to the brain and to bodily processes. These are due to micro-biological (cellular) processes, which in turn are due to biochemical processes and so on. But biochemistry or underlying physics are not enough and concepts - and the physical objects captured by them - such as “neurons,” “neural networks,” “activations” are essential. Indeed, they are a level of organization of a physical reality that possess new properties and dynamics.

An example of the very reductive outcome obtained by several attempts to establish the neural foundation of psychological (and social) notions is about the concept of “trust.”

As Fehr writes: “the rationale for the experiment originates in evidence indicating that oxytocin plays a key role in certain pro-social approach behaviors in non-human mammals. (...) Based on the animal literature, Kosfeld et al. (13), hypothesized that oxytocin might cause humans to exhibit more behavioral trust as measured in the trust game” (13, 14). In these experiments they also show how oxytocin has a specific effect on social behavior because it differently impacts on the trustor and the trustee (only in the first case there is a positive influence). In addition, it is also shown that the trustor's sensitivity to risk is not reduced as a general behavior but it depends on the partner nature (human vs. non-human). These are without any doubts very interesting data. However, the multidimensional and very articulated notion of trust (so crucial for individual feelings and conduct and for social relations) (15), should not be reduced to a generic pro-social attitude and to a particular chemical response or the mere activation of a given brain area. Trust is not a simple, vague, and unitary notion and disposition; it is made of rather complex evaluations, expectations, attributions, decisions to rely, sentiments. It should be a componential and analytical psychological model of trust to *drive* neural research rather than searching for a simplistic and direct solution, just localist and correlational (16).

Indeed, even the most accurate and complete knowledge of the micro-level does not allow us to infer structure, organization, dynamics, and “functions” of the macro-level. For instance, the explanation of cellular roles and activities and their laws cannot be reduced to the micro-description of their underlying chemical processes without losing necessary information. Cells are indeed implemented, founded on their chemical substrate and laws but we need the other layer of notions/concepts, their new functions, their laws [see also (17)]. Reduction is micro-foundation, material grounding, but not necessarily elimination.

Let us consider, for example, the following case: we want to evaluate whether a dancer correctly performs a certain dance step. Suppose it is possible to detect all changes in all of the dancer's muscles as she dances. Even if we have a computer with an enormous computational capacity, could we entrust the computer with the evaluation of her dance? That is, does the complete and accurate recording of the activation of the dancer's muscles allow for an aesthetic evaluation? No. For at least two reasons. The first is that we should also codify the parameters describing the muscle activation patterns relevant for the evaluation; an information which could otherwise not be inferred just by the sum of the data concerning the movements of the different muscles. The second is that we will also have to translate in a computational form the aesthetic criteria discriminating the activation patterns that characterize good executions; an information which is also not inferable just by muscle registration. In other words, we should enter into the computer information concerning the macro level and which cannot be inferred from the data coming from the micro level. It would be non-sense to pretend to understand if the movement of the dancer corresponds to aesthetic criteria, only by studying the movements of her muscles and without knowing the aesthetic criteria. And for those involved in dance, for example a choreographer, aesthetic criteria are indispensable.

It seems plausible that a (very large) machine learning model fed with enough labeled examples could be trained to reproduce a fair aesthetic assessment of a dance from a stream of pixels in a video. But on the condition of providing labeled examples of correct and incorrect movements, that is, examples of the application of aesthetic criteria that nevertheless belong to a different level from that of muscle movements. Aesthetic criteria can be reduced to movements but they are not necessarily deduced on the basis of movements. In other words, aesthetic criteria *supervene* on movements [for a definition of Supervenience and its distinction to emergence see (18)].

We do not think that the problem raised by eliminativism is just “practical” and one destined to be overcome as the knowledge about the brain advances.

Rather, we believe that *at the epistemological level* (i.e., in order to understand reality) another level of description of reality is needed and more specifically, the level of emerging macro-functions which define and model processes and mechanisms. Science should be modeling, conceptualization, description and explanation not just at the micro-micro level but also at the different functional levels of complexity. This does not involve a dualism of reality but a dualism of theory and concepts (as also in the physical and natural sciences: material vs. functional concepts, and not on two levels but on layers). Indeed, we assume that reality is one and material but we believe that, in order to understand it, we need to consider different levels of emergent properties that can be grasped with conceptual categories appropriate to that specific level and cannot be grasped otherwise (i.e., with categories belonging to a lower or upper level). For example, given that viruses are ultimately made of atoms and atoms of electrons, using just lower-level atomic-physics conceptual categories to understand how viruses work, does not appear substantially appropriate, because aspects that are crucial for the understanding of viruses, such as for example their architecture and methods of reproduction, are not captured by the lower level concepts of atomic physics. To answer these questions, the knowledge of the virologist is necessary, that is, a body of knowledge that grasps reality at a different level than that of atomic physics. Indeed, other conceptual categories are needed, and these are not only pragmatically more useful than those of the atomic physics; they are irreplaceable for understanding and explaining viruses as well as for acting on them. Those of the atomic physicists can contribute to enrich the knowledge of the virologist, but not replace them, as well as those of the epidemiologist and sociologist, who look at the phenomenon at even more macro levels, can complement those of the virologist but not replace them. Importantly, since we assume that reality is one, even if it can be described at different levels and from different points of view, it follows that we cannot strictly speak about “causality” between different levels. As suggested by Kim (19) psychophysical causal relations should be viewed as epiphenomenal supervenient causal relations (20). To understand this concept Kim (19) proposes the following example: “Thus, if a pain causes the sensation of fear an instant later, this account tells the following story: the pain is supervenient on a brain state, this brain state causes another appropriate brain state, and given this second brain state, the fear sensation must occur, for it is supervenient upon that brain state” (19). A mental event is not caused by a neural event since they are the same thing, described at two different levels, with different categories that are able to grasp the characteristic properties of one level but not the other. In this article, of the many possible levels, we are interested in two, i.e., the neural and the mental (e.g., not the molecular and not the social), it seems interesting to observe an asymmetry between the two.

While it is true that the characteristics of the macro-level cannot necessarily be inferred from the characteristics of the micro-level, the opposite is true. Inferences from the macro to the micro level are possible, and therefore the study of the micro-level could not only be used, but it should be used as a bench test for psychological hypotheses. It *should* because, if it is true that the mind is implemented in the brain, then any mental hypothesis must be compatible with the structure or functioning of the brain.

A research (21) tested the hypothesis, strictly psychological, that there are two types of guilt feelings, one altruistic and one deontological. Deontological guilt was induced in one group and altruistic guilt in another group of non-clinical participants. During the induction, brain activity was detected *via* fMRI. The results showed that the two guilt feelings have a different neural substrate. Therefore, the hypothesis has been corroborated. It should be noted that no CNS analysis, however accurate and exhaustive, could have made sense of the neural activation patterns detected in this study, had it not been accompanied by psychological assumptions. Furthermore, it cannot be overlooked that renouncing to the psychological construct of guilt would imply renouncing to explain and predict many behaviors and interactions between people. It is interesting to observe that from the aforementioned study, it emerged that deontological guilt, but not altruistic guilt, shares part of the neural substrate with disgust, specifically the insular cortex. These results might also explain another psychological problem which concerns the relationship between guilt and disgust in the so-called Macbeth effect, in which the induction of guilt increases disgust sensitivity and washing the body reduces guilt (22). The Macbeth effect has been found inconsistently in some studies but not in others. However, it becomes clear only if the type of guilt induced is deontological and not if it is altruistic (23). Taken together these studies well represent an example of the use of neural data to assess psychological hypotheses. Specifically, here H_1_ was that guilt can be conceptualized in two distinct emotional patterns and that these differences are also reflected in brain activity. Furthermore, the results also helped to clarify why the Macbeth effect was observed only in some studies but not in others. Indeed, previous research did not consider separately the effects of deontological and altruistic guilt.

In keeping, two behavioral studies have shown that induction of deontological guilt implies more thorough and prolonged washings than induction of altruistic guilt (24, 25), and two other studies, using transcranial direct current stimulation (tDCS), showed that a stimulation of the insular cortex implies an enhancement in disgust and orient moral judgments in a deontological sense, while the inhibition of the insula has the opposite effect. On the other hand, there is no effect on altruistic moral judgments (26, 27).

In a similar vein, some researchers observed that the dysfunction of the social brain in schizophrenia is modulated by intention type. Specifically, patients showed significantly less activation in three regions typically activated in ToM tasks, i.e., paracingulate cortex and bilateral temporo-parietal junctions. However, this dysfunction was present only for social but not for non-social intentions (28). In this case, neuroscientific findings helped to determine that also the psychological concept of “intention” can be differentiated on the basis of the object of the intention and that only certain types of intention are abnormal in schizophrenic patients.

An anonymous reviewer suggested that one could collect a large number of guilt instances and corresponding brain activation patterns, then run some kind of clustering to see if distinct grouping emerges; it is possible that such a micro → macro approach would reveal partially differentiated clusters of brain activity, which could then reveal corresponding differences in the corresponding guilt episodes. However, to carry out this operation of searching for differences between guilt feelings starting from the neural data collection it is necessary to have psychological categories, such as “guilt feelings,” and to define corresponding differences in the corresponding guilt episodes, such as the absence or presence of an affective relation between the guilty and the victim. Moreover, without the knowledge contained at the mental level, the neuroscientist might incur in the multiple realization problem (i.e., the thesis that the same mental state can be realized by different physical states), (29–31).

Mental representations, functions, and processing are just material, informational entities; emergent *functions*, described in informational/functional terms, but if they are brought back to their underlying micro-processes, they will not be redundant and eliminable. The psychological notions should be preserved for understanding and explaining “what the brain is *doing*”: perceiving, memorizing, retrieving, deciding, pursuing, and so on; at its emergent, macro-functional level of activity.

Neural correlates cannot be the right *vocabulary* for explaining human behaviors, just because they refer to concepts pertaining to the micro-level and do not represent and discriminate the complex “patterns” and their properties and functions (not of their sub-components) at the cognitive and motivational macro-level. Once we will have the real neural representation of a complex object like a “motivating goal,” or an “altruistic intention,” or of a real “trust attitude,” or a “complex emotion with its appraisal components” like envy, we will have a quasi-complete explanation of it, but we could not renounce to that psychological vocabulary^1^; since it holds at the functional/informational macro level (12).

More in general: there are no alternatives to the need for *reading* and *understanding* body in terms of functions, not just in terms of “simple” matter and its physico-chemical processes description. We look at the kidney as a “filter,” at glands in terms of “secretion.” Otherwise we do not understand what they do, that is, what they are; which is the sense of the physico-chemical processes that we are describing. Indeed, we know the world through its functions. Even the most basic categories (e.g., fruit, apples) are organized to give information on the functions of a certain element. In this way we also know biology or economics and so on. The same obviously holds for our brain. Neuroscientists shouldn't try to “skip” psychology and its information-processing models of structures and manipulations, for directly connecting brain with behavior (neuro-economics, neuro-aesthetics, neuro-ethics, neuro-politics,...). On the contrary they should take the procedural (possibly computational) models of the cognitive sciences and find their neural grounding or - if this proves unfeasible - change them. In fact, a cognitive model that is not grounded in our brain and somatic processes is just wrong, unacceptable. And - on the other side - psychology should provide model**s** of proximate processes; not just correlational “theories” (7, 12, 32, 34).

## Are the diseases we treat diseases of the brain?

Under what conditions can we consider this statement legitimate?

As is well known, the problem regarding the definition of psychopathology is still debated and concerns the possibility of basing the diagnosis on objective and non-evaluative criteria.

For instance, according to Christopher Boorse's biostatistical account, to define a (mental) disease value-laden judgements are not necessary: “if diseases are deviations from the species biological design, their recognition is a matter of natural science, not evaluative decision” (35). This definition holds for mental disorders on the condition that a definition of mental disorder is informed by our knowledge of biological design. Differently, Jerome's Wakefield *hybrid naturalism' a*ccepts a value component (harm), while still embracing an objective, evolutionary account of natural functions (36).

Here, we do not enter into the merits of this still unresolved debate on the definition of psychopathology, (i.e., on the criteria that differentiate psychopathology from normality). We simply base our definition of psychopathology on the DSM 5 or ICD 11. Indeed, rather than drawing a final conclusion about what psychopathology is or not, here we discuss the differences between psychopathology and neuropathology at the brain level.

Secondly, from neuroscience, for the moment, no criterion has emerged that allows a reliable psychiatric diagnosis, that is, without an exaggerated number of false positives and negatives, but, even if a neural marker is found as a valid diagnostic tool, would this justify such a conclusion?

The answer is necessarily articulated.

Let's consider an example of a psychopathological disorder underlying a brain disease: progressive paralysis. It is a serious neuropathological form caused by the treponema of syphilis which manifests itself, among other things, with mood changes and delusions. The symptoms are predominantly psychiatric and the cause is exclusively neurological and, specifically, infectious. Similarly, important psychopathological, emotional and behavioral alterations, up to real personality disorders, can be caused by traumatic, neoplastic, infectious or degenerative lesions of the frontal lobe. In these cases, the brains of patients are different from that of non-patients for their neuropathological characteristics. Here the mental disorder is underpinned by a true brain disease a true brain disease, in fact, there are characteristics of the CNS that are compatible with the anatomy and physiology typical of neurological diseases. In these cases, the statement of Ross and colleagues is justified.

There are other cases in which psychopathological disorders are accompanied by brain damage but which nevertheless do not justify Ross's conclusion. It is well known that the incidence of psychopathology in people with intellectual development disorder is higher than usual (37, 38).

It is plausible that at the basis of some forms of intellectual disability there is a brain damage due to infectious, neoplastic, metabolic, degenerative, autoimmune, traumatic or genetic causes. It is equally evident that the cognitive outcomes of these damages interact with psychological variables, for example with greater difficulty in regulating emotions, and with social variables, for example with social exclusion, which in turn interacts with self-esteem, producing psychiatric symptoms. Also, in this case there is a neurological damage, but the brain injury and its cognitive consequences are just a vulnerability factor to psychopathology and not the necessary and sufficient cause, as it happens in progressive paralysis. There are differences in the brain due to neuropathological alterations but these are not the cause of psychopathology, rather, their consequences represent a vulnerability factor for psychopathology. Let us now consider, for instance, the brain of a person suffering from OCD. With a certain approximation it can be said that his brain is anatomically and functionally different from that of other people (39), but not in the same way as in patients with progressive paralysis or with frontal injuries. In fact, the brains of patients with OCD do not show the typical signs of neurological diseases, in which neurons are abnormal with respect to the laws of neuroanatomy and neurophysiology, for instance, the electrical activity of an epileptic brain, the presence of beta amyloid plaques, demyelinated plaques or gliotic infiltrates. A similar consideration can be extended to synaptic mediators. For instance, some results suggest that the density of serotonin (5-HT) transporter ^3^H-Par binding sites was significantly lower in OCD patients than in controls. Could we infer from these data a damage in serotonin metabolism in OCD patients? Not necessarily, because the same alteration has been observed in people who are in love (40). Thus, the fact that the density of ^3^H-Par binding sites is significantly lower in OCD patients than in controls is not necessarily an expression of a brain disease unless we also claim that love is a brain disease. It would seem more correct to state that we are in the presence of normal variations of serotonin metabolism which are connected to different mental states.

Certainly, it cannot be excluded that, in the future, the knowledge of pathological anatomy and pathophysiology will increase, enabling us us to recognize signs of actual neuropathologies in the brain of obsessive patients, but at the moment it does not seem to be so, without prejudice to that nosographic entity (i.e., the Pediatric Autoimmune Neuropsychiatric Disorders, PANDAS) (41), whose existence is still debated and scarcely accepted by most and which in any case would concern a small subset of people with obsessive compulsive disorder. The differences that the brains of patients with OCD have to those of healthy controls is more similar to the differences found in the brains of “experts” (42). For instance, the brains of professional pianists are structurally different from that of other people but the neurons are not pathological, rather they are well functioning with respect to the laws of neuroanatomy and neurophysiology (43, 44).

Similarly, we can assume that a football fan has a different brain functioning than a person who is completely disinterested in football or a fan of an opposing team (45, 46). Even in this case we can speak of differences in terms of behaviors, assessments, and emotions, but we cannot say that the fan's brain is abnormal with respect to the laws of neurology. Let's now consider the case of a person that is moved not by the passion for the piano or for a football team but by the passion for cleaning, and they are an expert not in pianos and not even in playing schemes but in the prevention and neutralization of contamination. We can observe that her brain is different from that of other people. Now suppose a psychiatrist tells us that this person is suffering from Obsessive Compulsive Disorder, that is, from a psychopathology. Would this diagnosis be sufficient to affirm that the observed cerebral diversity is similar to that of the patient suffering from progressive paralysis or from lesions of the prefrontal lobe? No, unless we observe anatomo-functional abnormalities with respect to the neuropathological criteria that discriminate a healthy nervous system from a sick one, for example degenerative or neoplastic lesions, outcomes of trauma, signs of infection or autoimmune reactions. If these conditions are not met, then we are in the presence of the many individual differences that characterize every organ of the human body. It does not appear legitimate, therefore, to infer a disease of the brain just because a diversity is observed, even if the diversity observed in the brain corresponds to a psychopathology. If this limit is not admitted, there is a risk of a paradox. Let's see it. We can imagine, for the benefit of our argument, that the brain of a homosexual person is different from that of a heterosexual [extensive findings indeed suggest that human sexual orientation is associated with brain morphology, e.g., (47)].

Nowadays, no one would say that homosexuality is a form of psychopathology, therefore the observed diversity appears similar to that found in pianists: different interests, different ways of being that correspond to different brains.

Now, suppose we go back in time, to 70 years ago. Homosexuality was considered a form of psychopathology. Would this have implied that the diversity of the brains of homosexuals was analogous to that of the patient with progressive paralysis? That is, can brain diversity be neuropathological or cease to be so, only as a consequence of conventional decisions about what is or is not psychopathological^2^? Here it seems very pertinent what Protopapas and Parrila (49) write about the dyslexia: “… differences in brains are certain to exist whenever differences in behavior exist, including differences in ability and performance. Therefore, findings of brain differences do not constitute evidence for abnormality; rather, they simply document the neural substrate of the behavioral differences. We suggest that dyslexia is best viewed as one of many expressions of ordinary ubiquitous individual differences in normal developmental outcomes. Thus, terms such as “dysfunctional” or “abnormal” are not justified when referring to the brains of persons with dyslexia” (49).

A mental pathology does not necessarily imply a malfunction, an anomaly in the neural mechanisms in which it is implemented. A psychic malfunction does not imply a neural malfunction. To use the computer analogy, a software may not work, if it is poorly made or damaged, without any damage or problem to the hardware in which it is implemented. Similarly, a complex algorithm may not work well, even if the basic software in which it is written is perfect and works smoothly; it is that very algorithm to be faulty.

Of course, if a brain disease is there, there can be psychopathological repercussions. Similarly, if the hardware is damaged, the software and the algorithm might also not work properly. These examples portray well how there is a *non sequitur* between the (obvious) idea that dysfunctional / psychopathological processes are *brain processes* and the assumption that *therefore* their cause *must* be a brain damage, a neural or biochemical dysfunction, a neural *disease* (12).

## Psychotherapy and the brain

Similarly, there is a *non sequitur* between the (obvious) idea that dysfunctional/psychopathological (and recovery) processes are *brain processes* and the assumption that *therefore* the intervention must necessarily and *directly* be on the brain and its functioning [see also (50)].

To think something is a new state of our brain; to learn something is to modify our brain; to relearn, adjust previous learning, is to modify our brain again (12). There might have been, for several concurrent factors, a *dysfunctional* learning, dysfunctional thoughts, and the challenge is, restructuring the learned representations and processes, through new cognitive and affective experiences and mental elaborations. Any change in our conduct or attitudes is a change in our minds; any change in our minds is a change in our brains. Our brain has also been materially “written” by our conduct, experience, and environment. In psychotherapeutic, educational or rehabilitation interventions the challenge is to preserve this route, and this view. For changing our brain, we do not need to directly act on our brain. Similarly, for producing water we do not need (and it is even worst) to join oxygen and hydrogenous; or for changing genes regulation not necessarily we manipulate genes (epigenetics).

According to Karlsson (51): “Psychotherapy outcomes and the mechanisms of change that are related to its effects have traditionally been investigated on the psychological and social levels, by measuring changes in symptoms, psychological abilities, personality, or social functioning. Many psychiatrists have also held the unfortunate dichotomized position that psychotherapy is a treatment for “psychologically based” disorders, while medication is for “biologically based” disorders. During the past several decades, it has become clear that all mental processes derive from mechanisms of the brain. This means that any change in our psychological processes is reflected by changes in the functions or structures of the brain. Straightforward reductionistic stances, however, are unfounded because there is clear evidence that our subjective experiences affect the brain”.

Empirical and meta-analytical data have shown that:

Several types of psychotherapies modify the brain structure and its functioning. “…cognitive-behavioral therapy (CBT), dialectic behavior therapy (DBT), psychodynamic psychotherapy, and interpersonal psychotherapy alter brain function in patients suffering from major depressive disorder (MDD), obsessive-compulsive disorder, panic disorder, social anxiety disorder, specific phobias, posttraumatic stress disorder, and borderline personality disorder (BPD)” (51); these changes sometimes appear similar to those obtained with drugs and sometimes different. “The majority of these studies have reported similar brain changes after psychotherapy and medication. However, some recent studies have also shown clear differences among these treatment modalities” (51); sometimes psychotherapy modifies precisely the brain characteristics that are considered specific to a disorder. e.g., in depression, “Behavioral therapy for anxiety disorders was consistently associated with attenuation of brain-imaging abnormalities in regions linked to the pathophysiology of anxiety, and with activation in regions related to positive reappraisal of anxiogenic stimuli.,” and in OCD: “The symptoms of obsessive–compulsive disorder (OCD) include intrusive thoughts, compulsive behavior, anxiety, and cognitive inflexibility, which are associated with dysfunction in dorsal and ventral corticostriato-thalamocortical (CSTC) circuits” (52). Psychotherapy involving exposure and response prevention has been established as an effective treatment for the affective symptoms, 16 studies measuring neural changes after therapy were included in the review. Post-treatment decreases of symptoms and activity in the ventral circuits during symptom provocation, as well as mainly increased activity in dorsal circuits during cognitive processing. These effects appear to be common to both psychotherapy and medication approaches” (53). It could be argued that these changes are functional and not structural and that the latter may not be affected by psychotherapeutic interventions. However, some data suggest that prolonged psychological interventions can modify those structural aspects that are considered distinctive of a given psychopathological disorder. Some examples: “Research in recent decades has (…) *provided* compelling evidence that learning new behavior can alter the structure of the adult human brain” (42). This learning-dependent structural plasticity has been shown for psychotherapy. Two years of cognitive remediation therapy increased gray matter volume in the fusiform gyrus, hippocampus and amygdala (54) as well as fractional anisotropy in the genu of the corpus callosum in patients with schizophrenia (55). Ten weeks of cognitive behavioral group therapy reduced gray matter volume in parieto-occipital and prefrontal regions and increased fractional anisotropy in the uncinate and inferior longitudinal fasciculus and structural connectivity in a frontolimbic network in patients with social anxiety disorder (56). “We found that DBT increased gray matter volume of brain regions that are critically implicated in emotion regulation and higher-order functions, such as mentalizing. The role of the angular gyrus for treatment response may reside in its cross-modal integrative function. These findings enhance our understanding of psychotherapy mechanisms of change and may foster the development of neurobiologically informed therapeutic interventions” (57). Hoexter et al. (58) found that abnormalities in gray matter volume in the left putamen were no longer detectable after CBT. Finally, Zhong and colleagues, (59) found that white matter alterations in some regions (i.e., left orbital frontal cortex, right cerebellum, right putamen nucleus, which play an important role in the neural mechanisms of OCD) can be reversible following an effective course of CBT (58, 59).

These data lend themselves to two considerations. The first is that psychotherapy changes the brain. It is worth noting, that affirming this does not necessarily imply mental causation (a very complex and still debated problem) (60). Indeed, as pointed out by Davidson “each individual mental event is in fact a physical event in the following sense: any event that has a mental description has also a physical description. Further, it is only under its physical description that a mental event can be seen to enter into a causal relation with a physical event (or any other event) by being subsumed under a causal law” (61). Psychotherapy consists of an exchange of information that takes place through verbal and non-verbal channels, and since information is nothing more than energy, organized in different ways, but still energy, psychotherapy must have an impact on the brain, and ultimately on the atoms that compose it.

The second consideration is that the influence of psychotherapy on the brain is not non-specific but, as at least suggested by some research, it modifies aspects of the brain that are specifically involved in the psychological disorder which is being treated. It is important to note that this is different for instance from what happens through rehabilitation after a brain injury. For instance, a thrombosis in a cerebral artery is likely to cause the death of a group of neurons which will be substituted by glial cells. Let's imagine that this causes a functional damage, e.g., aphasia. The function of language can be restored through speech therapy, which thanks to neural plasticity, can modify the micro-anatomic organization of the brain, but it cannot repair the specific area of the brain that was damaged (i.e., its specific substratum), that is, it cannot turn glial cells back into neurons again. The difference with psychotherapy here consists in the observation that psychotherapy is able to change those same neural characteristics that are considered as proof of the putative neuropathological origin of those mental disorders. For instance, glucose metabolic rates in the right head of the caudate change when OCD is successfully treated with either fluoxetine or behavior therapy (62). This means that psychotherapy is able to restore the specific substratum of a psychopathological disorder, precisely because this substratum was never “damaged.”

If psychotherapy is able to change the specific substratum of a psychopathological disorder, then it is difficult to argue that “The diseases we treat are diseases of the brain,” only on the basis of the discovery of specific cerebral, functional and structural characteristics. If psychopathologies were true neurological diseases, such as Alzheimer's or multiple sclerosis or Huntington's chorea, their specific neural substrate would not be modifiable by psychotherapy. Indeed, it is not plausible that a psychotherapy can reduce the beta amyloid plaques in Alzheimer's disease, even if psychotherapy could reduce anxiety and depression reactive to the awareness of being affected by this serious disease.

## Conclusions

Interpreting the statement “The diseases we treat are diseases of the brain” in a literal way implies, in our opinion, two critical points. The first is the assumption of an eliminativist perspective, at least in the domain of psychopathology. Psychopathological manifestations would be devoid of intrinsic meaning and therefore would need an explanation at the neural level, a level that Dennett would define as “sub-personal” (63). Moreover, according to this perspective it would be useless, or even misleading, to try to explain psychopathology by resorting to the contents of the patient's mind, (i.e., his mental representations, his beliefs and his own goals); in other words, to use the explanation level which, according to Dennett, we could define as “personal.” In short, the statement “The diseases we treat are diseases of the brain” appears underpinned by an eliminativist reductionism that we here challenged by presenting arguments in favor of emergent reductionism.

The second point is the following. The differences found in the brains of people with psychopathology would be neuropathological differences, that is, abnormal with respect to the anatomical and physiological criteria that define the healthy brain. Here, we contested the idea that it is enough to find a difference between the brains of people suffering from psychopathology and that of people who are not affected or affected by different psychopathologies. We therefore disentangled between psychopathological disorders underlying a true pathology of the brain from those underlying simple anatomical or functional differences. Differences that are similar to those that are normally found between individuals, even among those who are not affected by psychopathologies. Finally, we considered some studies which show how purely psychological interventions can reduce or eliminate the differences between the brains of people with psychopathology and those of people without.

## Author contributions

All authors listed have made a substantial, direct, and intellectual contribution to the work, and approved it for publication.

## Funding

The publication of this work has been funded by the School of Cognitive Psychotherapy, SPC, Rome, Italy.

## Conflict of interest

The authors declare that the research was conducted in the absence of any commercial or financial relationships that could be construed as a potential conflict of interest.

## Publisher's note

All claims expressed in this article are solely those of the authors and do not necessarily represent those of their affiliated organizations, or those of the publisher, the editors and the reviewers. Any product that may be evaluated in this article, or claim that may be made by its manufacturer, is not guaranteed or endorsed by the publisher.

## Footnotes

^1^Let's also remark that the criticism of Elimitativism to Psychology, i.e., that psychology would just use common-sense words, would just be “folk psychology” without scientific notions, is false/wrong: consider for example the notion of “goal,” which is very contrary to common sense (with its notions of feedback, circular cusality, exc.) and it is directly derived from Cybernetics (32, 33).

^2^It should be noted that also in agreement with Wakefield and Conrad (48), in order to define psychopathology, an evaluation criterion is indispensable.“The HDA maintains that a disorder is a harmful condition-judged by social values, thus value laden-caused by a dysfunction, where “dysfunction” is a factual concept that refers to a failure of some feature of the organism to perform a natural function…”.
